# Acoustic NLOS Identification Using Acoustic Channel Characteristics for Smartphone Indoor Localization

**DOI:** 10.3390/s17040727

**Published:** 2017-03-30

**Authors:** Lei Zhang, Danjie Huang, Xinheng Wang, Christian Schindelhauer, Zhi Wang

**Affiliations:** 1 State Key Laboratory of Industrial Control Technology, Zhejiang University, Hangzhou 310027, China; zhlei0202@163.com (L.Z.); huangdanjie07@zju.edu.cn (D.H.); 2 School of Engineering and Computing, University of the West of Scotland, Paisley PA1 2BE, UK; xinheng.wang@uws.ac.uk; 3 Department of Computer Science, University of Freiburg, 79110 Freiburg, Germany; schindel@informatik.uni-freiburg.de

**Keywords:** NLOS identification, smartphone indoor localization, acoustic channel gain and delay, support vector machine (SVM), RBF kernel

## Abstract

As the demand for indoor localization is increasing to support our daily life in large and complex indoor environments, sound-based localization technologies have attracted researchers’ attention because they have the advantages of being fully compatible with commercial off-the-shelf (COTS) smartphones, they have high positioning accuracy and low-cost infrastructure. However, the non-line-of-sight (NLOS) phenomenon poses a great challenge and has become the technology bottleneck for practical applications of acoustic smartphone indoor localization. Through identifying and discarding the NLOS measurements, the positioning performance can be improved by incorporating only the LOS measurements. In this paper, we focus on identifying NLOS components by characterizing the acoustic channels. Firstly, by analyzing indoor acoustic propagations, the changes of acoustic channel from the line-of-sight (LOS) condition to the NLOS condition are characterized as the difference of channel gain and channel delay between the two propagation scenarios. Then, an efficient approach to estimate relative channel gain and delay based on the cross-correlation method is proposed, which considers the mitigation of the Doppler Effect and reduction of the computational complexity. Nine novel features have been extracted, and a support vector machine (SVM) classifier with a radial-based function (RBF) kernel is used to realize NLOS identification. The experimental result with an overall 98.9% classification accuracy based on a data set with more than 10 thousand measurements shows that the proposed identification approach and features are effective in acoustic NLOS identification for acoustic indoor localization via a smartphone. In order to further evaluate the performance of the proposed SVM classifier, the performance of an SVM classifier is compared with that of traditional classifiers based on logistic regression (LR) and linear discriminant analysis (LDA). The results also show that a SVM with the RBF kernel function method outperforms others in acoustic NLOS identification.

## 1. Introduction

**Figure 1:**
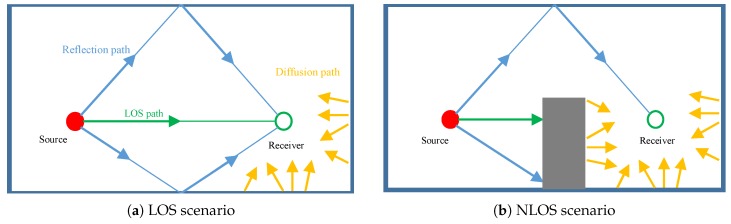
Line-of-sight (LOS) and non-line-of-sight (NLOS) scenario description.

As smart mobile devices have been ubiquitously available for people to use in our daily life, a new demand for indoor navigation, precision marketing, public safety and emergency rescue has emerged, especially in large buildings such as underground parking, large-scale transportation terminals, and large shopping malls [1]. Location-based services (LBS) using the conventional GPS system have been widely used in military and commercial sectors, but they are severely limited in indoor environments due to the strong attenuation of GPS signals [2]. In order to tackle the problems of indoor positioning, various approaches have been proposed by using the technologies based on sound, GSM, Bluetooth, Wi-Fi, light, and magnetic fields [3,4]. Among these approaches, sound-based positioning technologies have the advantages of being fully compatible with commercial off-the-shelf (COTS) smartphones, higher positioning accuracy than other technologies and low-cost infrastructure, and, thus, have attracted researchers’ attention. Quite a few systems have been designed and developed in the last decade in this area, aiming to introduce a reliable and practical technology for smartphone indoor localization [1,5,6,7,8,9]. However, from the results of Microsoft Indoor Localization Competition 2016 [10], the performance of sound localization systems is seriously impaired by indoor multipath propagation and the non-line-of-sight (NLOS) phenomenon in the real world. What is understood is that NLOS will introduce a significant amount of positive errors into target positioning, when the direct path between beacons and smartphones is blocked, as shown in Figure 1b. This will definitely degrade positioning accuracy and system stability. Therefore, the NLOS phenomenon poses a great challenge to the practical applications of acoustic smartphone indoor localization. It has already become the technology bottleneck which must be resolved to pave the way for the promotion of these technologies in the real world.

It is common that the line-of-sight (LOS) path, or direct path, is obstructed by human bodies, furniture, walls or corners, due to the arbitrariness of human movement. When LOS is not available, the received signals via NLOS will travel a longer distance than the LOS path. The estimation of the direction of arrival (DOA), time of arrival (TOA) and time difference of arrival (TDOA) would involve considerable errors. Through identifying and discarding the NLOS measurements, the positioning performance can be improved by incorporating only the LOS measurements [11,12,13]. Then, the measurements under the NLOS condition have to be identified.

The NLOS identification techniques for radio communications have been discussed extensively within cellular mobile networks and Ultra-Wideband (UWB) techniques, and many methods have been proposed [14,15]. These methods are based on ranging statistics [16,17], consistency among multiple measurements [18], and channel characteristics [19,20,21,22]. However, for acoustic NLOS identification, the research is still in its infancy, and only little pioneering research work has been reported. In underwater localization, Roee Diamant, Hwee-Pink Tan and Lutz Lampe identify object related NLOS links by comparing signal strength-based and propagation delay-based ranging measurements [23], but the acoustic NLOS identification in indoor environment is still an open problem.

Compared with wireless localization, the main characteristics of acoustic smartphone indoor localization are the low update rate of user positioning [9] and the poor consistency of sensor performance. This makes the methods mentioned above not suitable or challenging to use in order to address the acoustic NLOS identification via smartphones. For ranging statistics-based methods, it is very hard to obtain a set of historical range measurements in a small range and a short time-frame, due to low update rate. This method loses its data foundation. Regarding the methods based on consistency among multiple measurements: First, the one which compares the consistency between the DOA and direction of departure (DOD) cannot be used for smartphones. Second, when we use TOA and received signal strength (RSS) as the comparing pair, the consistency of performance among different sensors is very hard to guarantee, because the MEMS microphone and speaker of different COTS smartphones have different power magnification factors and frequency responses. This could severely degrade the identification performance.

**Figure 2:**
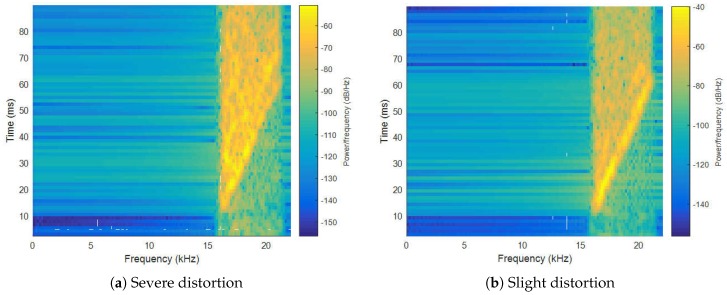
The distortion of received signals.

The methods based on channel characteristics are more suitable to address this problem. NLOS is induced by ambient environment, and the acoustic channel characteristics are also highly related to ambient environment, which makes using acoustic channel characteristics extracted from received signals a more direct way to realize NLOS identification. At the same time, the methods based on channel characteristics are a single-node approach which only uses the information of signals received from a single node. This could realize an independent and real-time acoustic NLOS identification of each ranging measurement between a transmitter and a receiver, and perfectly fit the acoustic indoor localization systems. However, many challenges still need to be overcome to realize acoustic NLOS identification via smartphones, including the following:

(1) The distortion of acoustic signals received by smartphones. It is understood that the MEMS microphone and speaker equipped in COTS smartphones are used for communication and entertainment. Once these modules are used as sensors for ranging measurement, many defects will be exposed. Except the poor performance and non-consistency of MEMS microphones and speakers, the speed of the crystal oscillator in smartphones, which provides the clock of the audio sampling and broadcasting system, is usually unstable. This could induce severe signal distortions, as shown in Figure 2. A linear-frequency-modulation (LFM) signal with 50 ms time duration, whose frequency band is from 16 kHz to 21 kHz, is broadcast by two Google nexu4 phones, and received by another same type of smartphone. We can clearly see that the signal in Figure 2a is severely distorted by the unstable sampling rate and Digital-to-Analogue Conversion (DAC) clock, while the signal in Figure 2b is slightly distorted. This phenomenon poses a great challenge for acoustic identification.

(2) The Doppler Effect caused by human movement. The Doppler Effect is another great challenge to acoustic NLOS identification, because smartphones are usually carried by human beings. The arbitrary movement of a human being coupled with arm swing makes the smartphone an extremely complex manoeuvring movement with a high speed. It could introduce an obvious shift of phase even at a slow walking speed, due to the low speed of sound propagation. Thus, a channel parameter estimation algorithm with the Doppler Effect mitigation is crucial for acoustic NLOS identification.

To the best of our knowledge, no prior works have considered and investigated LOS and NLOS identifications using the channel information from received acoustic signals in indoor environment. Therefore, aiming to address acoustic NLOS identification for smartphone indoor localization, we will systematically study this issue in this paper. The main contributions of this paper are as follows:

- An acoustic NLOS identification approach based on acoustic channel characteristics is proposed for smartphone indoor localization in the real world. This approach is suitable for the acoustic localization systems based on DOA, TOA and TDOA strategies.
- An efficient approach to estimate relative channel gain and delay based on the cross-correlation method is proposed, in order to mitigate the influence of the Doppler Effect and reduce the computational complexity.
- The differences and characteristics of acoustic relative channel gain and delay under LOS and NLOS conditions are investigated through extensive measurements in office rooms and lobby environment using COTS smartphones. Novel features are extracted from these characteristics that capture the salient properties based on time delay characteristics, waveform characteristics, Rician K-factor and frequency characteristics of relative channel gain.
- An optimal kernel function for an SVM classifier to realize acoustic NLOS identification is evaluated and chosen under the accuracy criterion, based on a data set with more than 10 thousand measurements. The best feature set of the SVM classifier for acoustic NLOS identification is investigated and proposed.

The remainder of the paper is organized as follows. In Section 2, we discuss the indoor acoustic propagation under LOS and NLOS conditions, and characterise the changes of acoustic channel from the LOS condition to the NLOS condition. In Section 3, an algorithm for estimating the acoustic relative channel gain and delay is introduced. The features extraction is described in Section 4. In particular, an acoustic signal acquisition method and an experimental environment are also introduced in this section. In Section 5, the SVM classifier and evaluation criteria are briefly introduced. The optimal kernel function and best feature combination are also given through cross-validation tests. At last, we draw our conclusions in Section 6.

## 2. Characterization of the Acoustic Channel under LOS and NLOS Conditions

Indoor environments are very complicated and different from each other. It is a dynamic environment due to the random walking of human beings and the displacement of small objects. In such a complicated environment, utilizing wave propagation theory, reverberation theory or a diffusion model to model indoor acoustic propagation is becoming difficult and complex. Geometrical room acoustics theory is a simplified model of indoor acoustic propagation [24]. In this theory, the sound wave is considered as a sound ray, just like the light, by employing the assumption that the dimension of the room and walls is larger than acoustic wavelength. The particularly important law of room acoustic is reflection. The refraction and curvature do not occur. Diffraction phenomena are neglected. Interference between multiple sound components is not considered. Then, it can be concluded that (1) the received signals consist of multiple components which are the copies of source signal with different power and time delay; (2) the power of the received signal comes from acoustic reflection and diffusion, and the reflection component represents a significant proportion.

### 2.1. The Characteristics of Room Acoustic Propagation under LOS Condition

For a signal s(t) broadcast from a speaker, the indoor propagation mainly includes LOS propagation, reflection and diffusion, as shown in Figure 1a. The signal x(t) received from these propagation paths can be expressed as

(1) $$x(t)=\sum_{l=1}^{n_{l}}H_{l}\left(s\left(t\right),\alpha_{l},\tau_{l}\right)+\sum_{r=1}^{n_{r}}H_{r}\left(s\left(t\right),\alpha_{r},\tau_{r}\right)+\sum_{d=1}^{n_{d}}H_{d}\left(s\left(t\right),\alpha_{d},\tau_{d}\right),$$

where the subscripts l,r and *d* denote the parameters related to LOS, reflection and diffusion paths, respectively, and H(·) represents the *n*th channel response with the path gain α and path delay τ. The characteristics of each kind of path are as follows:

- $n_{l}=\left\{1,0\right\}$. There is only one direct path between the transmitter and receiver, which is the LOS path. $n_{l}=1$ is the LOS condition, and 0 for the NLOS condition. $\alpha_{l}$ and $\tau_{l}$ are decreased with the increase of path length, due to the air propagation attenuation.
- The length of the reflection path is definitely longer than the LOS path. With the increase of reflection time, $\tau_{r}$ becomes larger and larger, while $\alpha_{r}$ is quickly decreased due to the acoustic absorption by air, walls and furniture. For the diffusion propagation path, the number of diffusion paths is usually very large. $\alpha_{d}$ and $\tau_{d}$ are related to the shape of the diffusion surface, absorption coefficient, and the relative position between the transmitter, receiver and diffusion surface.
- Generally speaking, the energy of signals received from the LOS path and reflection path is larger than the signals received from the diffusion path, that is $E_{l}\left(t\right)$, $E_{r}\left(t\right)$ > $E_{d}\left(t\right)$. However, the relationship between $E_{l}\left(t\right)$ and $E_{r}\left(t\right)$ is determined by ambient environment. It is common that the LOS signal is not the strongest, especially in large space environment.

### 2.2. The Characteristics of Acoustic Propagation under NLOS Condition

As shown in Figure 1b, when we put an object in the path between the transmitter and receiver, the LOS path disappears, which leads to the NLOS condition. Then, the LOS path and some short-length reflection paths totally disappear. At the same time, some long-range reflection paths emerge with the increase of reflection surfaces of blocking objects. Compared with the LOS scenario, the average length of the reflection path is definitely increased. Due to the increase of diffusion surfaces, the number of diffusion paths and the total signal energy of $x_{d}\left(t\right)$ are relatively increased.

Then, the changes of channel characteristics from the LOS condition to the NLOS condition include (1) the total energy of received signals is decreased; (2) the path gain of reflection paths is decreased; (3) the path delays of reflection paths and diffusion paths are all increased; (4) the relative proportion of diffusion signals is increased. All these changes could be characterized as the differences of the channel gain and channel delay between the LOS and NLOS propagation scenarios.

## 3. The Relative Channel Gain and channel Delay Estimation

As mentioned above, the changes, when an NLOS condition occurs, could be characterized as the differences of channel gain and channel delay between the LOS and NLOS propagation scenarios. Based on these characteristics, the features can be studied and extracted for acoustic NLOS identification. The research of acoustic channel parameter estimation is mainly conducted in underwater communications and the method based on Fractional Fourier Transform (FrFT) is widely used [25]. In order to mitigate the influence of the Doppler Effect and reduce the computational complexity, an efficient approach to estimate the relative channel gain and channel delay based on cross-correlation is proposed in this section. In an ideal condition, the channel impulse response (CIR) of room acoustics, denoted as h(t), can be expressed as

(2) $$h\left(t\right)=\sum_{i}\alpha_{i}\delta \left(t-{\bar{\tau }}_{i}\right),$$

where $\alpha_{i}$ and ${\bar{\tau }}_{i}$ are the path attenuation coefficients, also called the path gain and path delay, respectively. In order to estimate these two parameters, using a wide-band acoustic signal such as a UWB signal to measure the CIR is a direct way. However, the wide-band acoustic signal could introduce noise pollution to daily life. In addition, it is very hard to discriminate the TOA of the first arrival path due to the heavy background noises. Then, a modulated signal is more suitable for acoustic smartphone indoor localization and estimation of channel gain and channel delay.

### 3.1. Modelling of Received Signals

Using a speaker to broadcast an ideal modulated acoustic signal y(t), the complex form of the transmitted acoustic signal, or source signal s(t), is expressed as

(3) $$s\left(t\right)=y\left(t\right)*g\left(t\right)=A\left(t\right)e^{j(wt+\varphi_{0})},$$

where A(t), *w*, and $\varphi_{0}$ are the time domain weighting function, frequency and initial phase, respectively; the operator * is the convolution operation, and g(t) is the impulse response of the speaker. Then, the complex form of the received signal x(t), transmitted over an *L* path fading channel, can be written as [26]

(4) $$x\left(t\right)=s\left(t\right)*h\left(t\right)=\sum_{i=1}^{L}\alpha_{i}\left(t\right)A\left(t-{\bar{\tau }}_{i}\left(t\right)\right)e^{j[w\left(t-{\bar{\tau }}_{i}\left(t\right)\right)+\varphi_{0}+\varphi_{i}\left(t\right)]}+N_{i}\left(t\right),$$

where $\varphi_{i}\left(t\right)$ is the phase term of the Doppler Effect caused by the movement between the transmitter and receiver; $N_{i}\left(t\right)$ are the noises corresponding to each propagation path, which include Gaussian noise $N_{gi}\left(t\right)$ and non-Gaussian colored noise $N_{ci}\left(t\right)$. In this paper, we consider the distorted part of the signal as a kind of colored noise that has a strong energy and is closely correlated with the source signal.

Considering that the sound is a kind of low speed wave, the relative movement velocity between the transmitter and receiver caused by human beings is not a constant, and the parameter of environment also varies with time such as temperature, humidity and air pressure; the path gain $\alpha_{i}\left(t\right)$, path delay ${\bar{\tau }}_{i}\left(t\right)$ and phase term $\varphi_{i}\left(t\right)$ are all time-varying parameters. However, the time duration of each measurement is usually less than one second, which means the parameters of environment could be considered as constant or slow-varying values within such a short time-frame. Meanwhile, the length of the propagation path in indoor environment is usually short. Then, the path gain and path delay could be approximated as constants, i.e.,

(5) $$\alpha_{i}\left(t\right)=\alpha_{i}+\alpha_{i}^{{}^{\prime }}\left(t\right)\approx \alpha_{i},$$

(6) $${\bar{\tau }}_{i}\left(t\right)={\bar{\tau }}_{i}+{\bar{\tau }}_{i}^{{}^{\prime }}\left(t\right)\approx {\bar{\tau }}_{i},$$

where $\alpha_{i}$ and ${\bar{\tau }}_{i}$ are the constant components of the path gain and path delay, respectively. However, the approximation approach is not suitable for the phase term $\varphi_{i}\left(t\right)$, due to the time-varying characteristics of $\varphi_{i}\left(t\right)$ being more significant than the other parameters.

Since smartphones are usually carried by human beings, the arbitrary movement of a human being coupled with arm swing makes the smartphone an extremely complex manoeuvring movement with a high speed. This could introduce an obvious shift of phase even at a slow moving speed, due to the low speed of sound propagation. However, we can still divide $\varphi_{i}\left(t\right)$ into a constant part $\varphi_{i}$ and a time-varying part $\varphi_{i}^{{}^{\prime }}\left(t\right)$. Then, Equation (4) can be rewritten as follows:

(7) $$\begin{array}{ccc} x(t) & = & \sum_{i=1}^{L}\alpha_{i}e^{j\varphi_{i}^{{}^{\prime }}\left(t\right)}A\left(t-{\bar{\tau }}_{i}\right)e^{j[w\left(t-{\bar{\tau }}_{i}+\frac{\varphi_{i}}{w}\right)+\varphi_{0}]}+N_{gi}\left(t\right)+N_{ci}\left(t\right)\\ & \approx & \sum_{i=1}^{L}\alpha_{i}^{{}^{\prime }}s\left(t-{\bar{\tau }}_{i}^{{}^{\prime }}\right)+N_{gi}\left(t\right)+N_{ci}\left(t\right), \end{array}$$

where $\alpha_{i}^{{}^{\prime }}=\alpha_{i}e^{j\varphi_{i}^{{}^{\prime }}\left(t\right)}$ and ${\bar{\tau }}_{i}^{{}^{\prime }}={\bar{\tau }}_{i}-\varphi_{i}/w$. The impact of the Doppler phase term could be approximated to a low frequency carrier and an excess time delay. The constant part introduces a negative bias to the path delay, while the time-varying part is a multiplicative factor of the path gain. The existence of this term and the colored noises could bring a significant effect to the channel gain and delay estimation, and, at the same time, to the discrimination of the weak first arrival path. It has to be mitigated during the process of estimating the channel gain and delay.

### 3.2. Estimation Approach

As the Doppler phase term gives an excess product term to $\alpha_{i}$ and an addition term to ${\bar{\tau }}_{i}$, the channel parameter estimation problem could be formulated as the estimation of the relative path gain $r_{i}$ and relative path delay $\tau_{i}$ to mitigate its effects, which is expressed as

(8) $$\left\{\begin{array}{c} r_{i}=\frac{\alpha_{i}^{{}^{\prime }}}{\alpha_{m}^{{}^{\prime }}}=\frac{\alpha_{i}}{\alpha_{m}}e^{j[\varphi_{i}^{{}^{\prime }}\left(t\right)-\varphi_{m}^{{}^{\prime }}\left(t\right)]}\\ \tau_{i}={\bar{\tau }}_{i}^{{}^{\prime }}-{\bar{\tau }}_{1}^{{}^{\prime }}={\bar{\tau }}_{i}-{\bar{\tau }}_{1}+\frac{\varphi_{i}-\varphi_{1}}{w} \end{array}\right.,$$

where *i*=1 denotes the first arrival path and *i*=*m* denotes the path that has the strongest signal energy. $\left\{\left(r_{i},\tau_{i}\right);i=1,2,...,L\right\}$ is composed of the relative channel gain–delay set. Within a short time-frame, $(\varphi_{i}-\varphi_{1})/w\to 0$ and $e^{j[\varphi_{i}^{{}^{\prime }}\left(t\right)-\varphi_{m}^{{}^{\prime }}\left(t\right)]}\to 1$. Through this method, the influence of the Doppler phase term could be maximally mitigated, even eliminated when the relative moving speed between the transmitter and receiver is constant.

One of the most efficient estimators of relative channel gain and delay is based on the cross-correlation method. For the received signal x(t), we use an ideal signal y(t) as its reference signal because the source signal s(t) cannot be exactly obtained. Applying the cross-correlation method, the result is

(9) $$\begin{array}{ccc} R_{xy}\left(\tau \right) & = & \sum_{i=1}^{L}\int_{-\infty }^{+\infty }\alpha_{i}^{{}^{\prime }}s\left(f\right)y^{*}\left(f\right)e^{-j2\pi f{\bar{\tau }}_{i}^{{}^{\prime }}}e^{j2\pi f\tau }df+\int_{-\infty }^{+\infty }N_{i}\left(f\right)y^{*}\left(f\right)e^{j2\pi f\tau }df\\ & = & \sum_{i=1}^{L}\alpha_{i}^{{}^{\prime }}R_{sy}\left(\tau \right)*\delta \left(\tau -{\bar{\tau }}_{i}^{{}^{\prime }}\right)+R_{N_{ci}y}\left(\tau \right), \end{array}$$

where $R_{sy}\left(\tau \right)$ is the cross-correlation result of s(t) and y(t), and $R_{N_{c}iy}\left(\tau \right)$ is the result of colored noises $N_{c}\left(t\right)$ and y(t). Since s(t) cannot be precisely obtained, we could discuss the properties of $R_{sy}\left(\tau \right)$ as follows:

(1) If s(t) is identical to y(t) after both energy normalization, $R_{sy}\left(\tau \right)$ could be considered as the auto-correlation result. Then $R_{sy}\left(\tau \right)\leq R_{sy}\left(0\right)$.

(2) If s(t) approximates to y(t) after both energy normalization, then $R_{sy}\left(\tau \right)\leq R_{sy}\left(\rho \right)$, where ρ is a small constant value which is determined by the difference between s(t) and y(t). Therefore, in the interval $\left|\tau -\tau_{i}^{{}^{\prime }}\right|\leq \rho$, a positive extremum will definitely appear at the peak envelope of $R_{xy}\left(\tau \right)$. Thus, the estimated path delay ${\hat{\bar{\tau }}}_{i}$ can be calculated by

(10) $${\hat{\bar{\tau }}}_{i}=\underset{\tau }{Extremum}\left\{peaks\left[\left|R_{xy}\left(\tau \right)\right|\right]\right\},i=1,2,...,L,$$

where peaks[·] is the peak finding operator, and Extremum{·} is the extremum extraction operator. The value of $R_{xy}\left(\tau \right)$ at $\tau ={\hat{\bar{\tau }}}_{i}$ is

(11) $$\begin{array}{ccc} R_{xy}\left({\hat{\bar{\tau }}}_{i}\right) & = & \alpha_{i}^{{}^{\prime }}R_{sy}\left(0\right)+\sum_{j=1,j\neq i}^{L}\alpha_{j}^{{}^{\prime }}R_{sy}\left({\hat{\bar{\tau }}}_{i}-{\bar{\tau }}_{j}^{{}^{\prime }}\right)+\sum_{i=1}^{L}R_{N_{ci}y}\left(\tau \right)\\ & = & \alpha_{i}^{{}^{\prime }}R_{sy}\left(0\right)+R\left({\hat{\bar{\tau }}}_{i}\right)\;, \end{array}$$

where $R({\hat{\bar{\tau }}}_{i})$ is a residual term including the summation of adjacent path interference and the colored noise correlation term. Then, the estimated relative path gain ${\hat{r}}_{i}$ and relative path delay ${\hat{\tau }}_{i}$ can be calculated by

(12) $$\left\{\begin{array}{c} {\hat{r}}_{i}=\frac{\alpha_{i}^{{}^{\prime }}}{\alpha_{m}^{{}^{\prime }}}=\frac{R_{xy}\left({\hat{\bar{\tau }}}_{i}\right)-R\left({\hat{\bar{\tau }}}_{i}\right)}{R_{xy}\left({\hat{\bar{\tau }}}_{m}\right)-R\left({\hat{\bar{\tau }}}_{m}\right)}\approx \frac{R_{xy}\left({\hat{\bar{\tau }}}_{i}\right)}{R_{xy}\left({\hat{\bar{\tau }}}_{m}\right)}\\ {\hat{\tau }}_{i}={\hat{\bar{\tau }}}_{i}-{\hat{\bar{\tau }}}_{1} \end{array}\right..$$

In practical applications, the energy threshold method is commonly used to estimate the time delay of the first arrival path, which can be given by

(13) $${\hat{\bar{\tau }}}_{1}=\underset{{\hat{\bar{\tau }}}_{i}}{\arg\min}\left(R_{xy}\left({\hat{\bar{\tau }}}_{i}\right)\geq p_{thd}R_{xy}\left({\hat{\bar{\tau }}}_{m}\right)\right)\;,$$

where $p_{thd}\in \left(0,1\right]$ is the coefficient of energy threshold and depends on the signal to noise ratio (SNR). In this paper, we choose $p_{th}=0.3$ from experimental evaluations.

From Equation (12), by using the cross-correlation method, we can quickly calculate the relative channel gain and delay from received signals with a strong tolerance to the Doppler Effect. The processes are (1) applying the cross-correlation algorithm to the received signal x(t) with the ideal signal y(t) as the reference signal; (2) normalizing the amplitude of cross-correlation result $R_{xy}\left(\tau \right)$; (3) picking up the extremums of the peak envelope; (4) setting the first arrival path as the start time of the received signal. Then, the amplitude of the extremums is the estimated relative path gain ${\hat{r}}_{i}$, while the arrival time of the extremums is the estimated relative path delay ${\hat{\tau }}_{i}$. The data set $\left\{\left({\hat{r}}_{i},{\hat{\tau }}_{i}\right);i=1,2,...,L\right\}$ is the estimated relative channel gain–delay set. Based on the obtained relative channel gain and delay, some novel features can be extracted for acoustic NLOS identification.

## 4. Data Acquisition and Features Extraction

The data set of acoustic signals used in this paper is obtained by a series of experiments in office rooms and a lobby, respectively. The measurements are based on a non-invasive LFM audio signal, the frequency band of which is between 16 kHz and 21 kHz. The audio signal is broadcast and received by COTS smartphones in order to decrease the cost of infrastructure and make the experiments more general. The primary purpose is to characterize the effects of obstructions. By using currently available smartphones, we can quickly build an experiment platform by installing a specially developed Android application. Six smartphones are used for signals acquisition, that is two new HUAWEI Honor 4 (Huawei, Shenzhen, China) and four Google Nexus 4 (Gooogle, Mountain View, CA, USA) which had been used for 2 years. The frequency response test results of those two kinds of smartphones are similar to the results reported in [1]. In frequency bands lower than 8kHz, the frequency response shows a good linear characteristic, but decreases rapidly with the increase of audio frequency, especially when the audio frequency is more than 15 kHz. This phenomenon implies that the energy of the received acoustic signal between 16 kHz and 21 kHz could be sharply decreased. The radiation of the speaker in COTS smartphones shows a good omni-directional characteristic [1]. When the smartphones are placed on the tripod or attached on the wall and ceiling, we should pay attention to the location of the speaker installed in the smartphones, and make sure that the speaker has not been blocked.

### 4.1. Experiment Deployment

**Figure 3:**
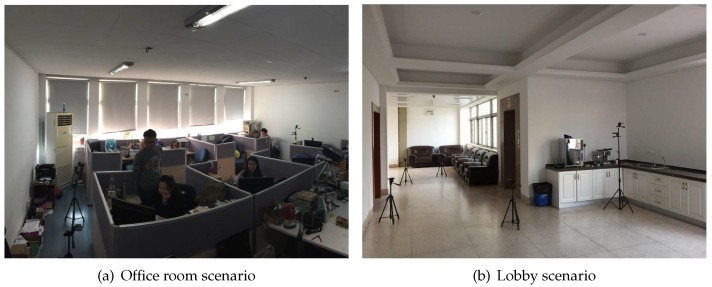
The measurement environment of the office room and lobby.

The primary purpose of the experiment is to characterize the effects of obstructions in office rooms and the lobby. Several office rooms and one lobby constitute this experiment, as shown in Figure 3. Those scenes are located in the New Industrial Control Building of Zhejiang University. The background noise intensity is between 50 dB and 65 dB. While the experiment is conducted in those particular environments through a large number of measurements and a variety of propagation scenarios encountered, we expect that the results are applicable in other office rooms and lobbies with similar environments.

#### A. Obstructions

**Figure 4:**
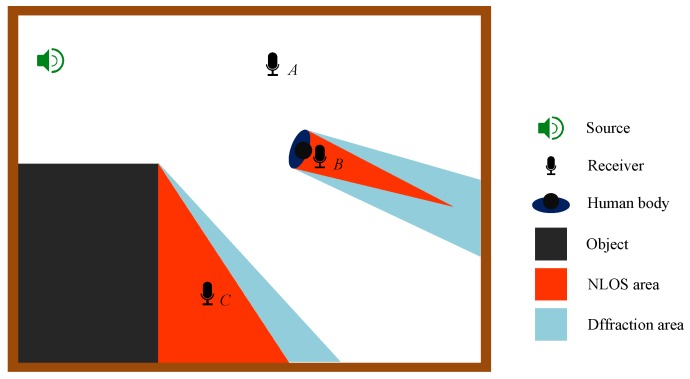
NLOS areas and diffusion areas.

Considering the actual NLOS condition, the obstructions include furniture, human body and corners. Even though we use the geometric room acoustic theory to describe room acoustic propagation for the sake of simplification, the diffraction phenomenon is actually existing. A brief depiction of this phenomenon is shown in Figure 4. The receiver deployed in the areas that are denoted as the diffraction area could receive a strong diffraction signal. The bias of range measurement in these areas is small enough to be considered as measurement noise. Thus, these areas could be classified into the LOS condition. In this situation, during the process of data acquisition under the NLOS condition, we avoid placing the receivers in those areas, since the boundaries of those areas are closely related to the shape and size of the room, and are very difficult to demarcate. Especially when we use the human body as an obstruction, the smartphone should be closely attached to the front or back of the human body, in order to make sure that the smartphone is deployed in red-colored areas, NLOS areas, where the diffraction components cannot be received.

#### B. Experiment Process

Since the reflection and diffusion of indoor acoustic propagation is a directional distribution, the displacement of acoustic sources could significantly change the sound field distribution. In order to extensively study acoustic propagations, we should measure sound signals where the transmitters are placed at different positions. The height of the receivers is fixed at 0.8 m, which is lower than the possible height held by a human hand in the standing pose, because a lower height means a higher obstructed chance and it is beneficial for quick data collection. The height of transmitters includes 0.8 m, 1.5 m and 2.2 m, respectively. All the smartphones are placed on tripods, in order to conveniently adjust the height and positions.

For the convenience of labeling the collected audio signals, the audio signals under LOS and NLOS conditions are collected separately. The process of the experiments is as following: (1) moving two acoustic sources to designated positions, and adjusting the height to 0.8 m; (2) dividing the measurement area into LOS and NLOS; (3) placing the four receivers at designated positions under the LOS condition; (4) moving the receivers to the next position with the displacement distances being limited at 0.2 m; (5) after measuring all the positions under the LOS condition, adjusting the height of sources to 1.5 m and 2.2 m, respectively, and repeating the processes (2)~(4) under the LOS condition; (6) moving two acoustic sources to the next designated position, and repeating the processes (1)~(5); (7) repeating the processes (1)~(6) for acoustic signals collection under the NLOS condition.

During the data collection process under the LOS condition, no human behaviors are forbidden in the measurement area except walking through and construction activities. The common office ambience sound has no influence on the measurements, such as music, steps, human voice and etc., since it could be easily filtered out by an FIR (Finite Impulse Response) high-pass filter. However, the impulse noise generated by construction activities, such as the sounds of pneumatic hammers and air nailers, could introduce severe spectrogram pollution to received signals in the considered high frequency band. At the same time, when a human being walks through the measurement area, it is very hard to label the condition of current measurement. However, under the NLOS condition, to simulate the dynamic status in the actual scenario, the human walk is necessary in the measurement area. In addition to that, one receiver is carried by a person to move around in NLOS areas to collect the audio signals corrupted by the Doppler Effect. Through those processes, more than 1000 positions are measured in each room and lobby. The size of the data set used in this paper is more than 10 thousand measured positions.

### 4.2. Features Extraction

**Figure 5:**
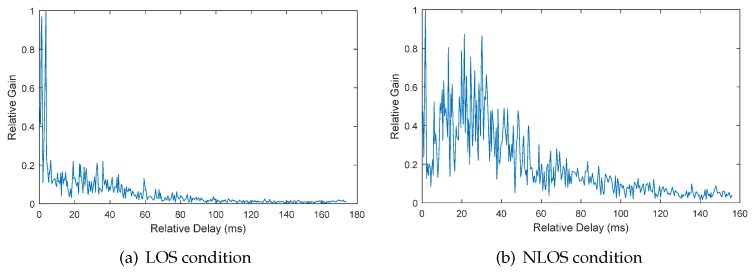
The relative channel gain and delay in the office room environment.

**Figure 6:**
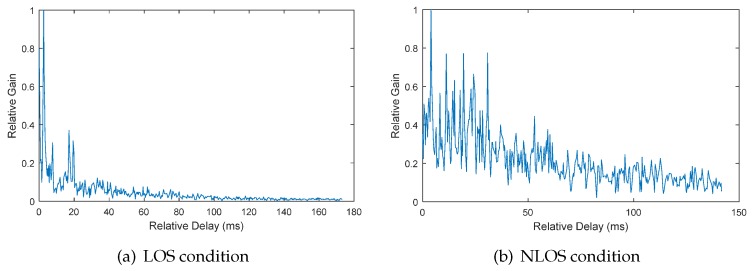
The relative channel gain and delay in the lobby environment.

Utilizing the approach proposed in Section 3.2, we can obtain the relative channel gain and delay of each acoustic signal in the data set. Shown in Figure 5 and Figure 6 are the typical channel gain and delay of LOS and NLOS conditions, respectively, in office rooms and the lobby. From the waveform, we can clearly see the difference between the two conditions. The main components under the LOS condition mainly concentrate on the early arrival time. However, the main components under the NLOS condition are more complex and mainly concentrated on the later arrival time. To characterize these differences, nine features are extracted. Corresponding to the changes when the NLOS condition occurs, which has been discussed in Section 2, the features based on time delay and waveform characteristics are firstly extracted. Referring to the Rician fading distribution of the wireless communication channel, the Rician K-factor is calculated as another kind of feature. The last kind of feature is based on the differences between the frequency distribution of relative channel gain in both conditions.

(1)  Time delay characteristics

The mean excess delay $\tau_{med}$ and Root Mean Square (RMS) delay spread $\tau_{rms}$ are the two statistics of delay spread, which could characterize the delay information to measure the multipath richness in the acoustic channel. The mean excess delay and RMS delay spread are, respectively, given by

(14) $$\tau_{med}=\frac{\sum_{i=1}^{L}{{\hat{r}}_{i}}^{2}{{\hat{\tau }}_{i}}^{2}}{\sum_{i=1}^{L}{\hat{r}}_{i}^{2}},\;\tau_{rms}=\sqrt{\frac{\sum_{i=1}^{L}{{\hat{r}}_{i}}^{2}{{\hat{\tau }}_{i}}^{2}}{\sum_{i=1}^{L}{\hat{r}}_{i}^{2}}-\tau_{med}^{2}}\;.$$

**Figure 7:**
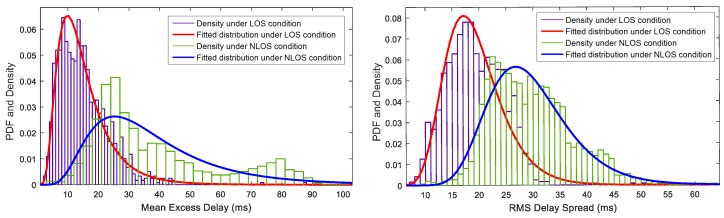
PDFs of the mean excess delay and RMS delay spread.

Generally, the values of $\tau_{med}$ and $\tau_{rms}$ under the NLOS condition are larger than those under the LOS condition. It can be explained as follows: (1) As the LOS path disappears, the first arrival path signal turns into a reflection path signal that usually has a lower energy; (2) The shortest reflection path also disappears. The average reflection path length is relatively increased, which also increases the time delay of the reflection path with a strong signal correspondingly; (3) The total energy of the received signal is decreased. Then, the proportion of the paths with small channel gain is relatively increased; (4) The additional diffusion surfaces of obstructions could increase the power and the time duration of the diffusion process. Thus, compared with the LOS condition, the values of $\tau_{med}$ and $\tau_{rms}$ are larger under the NLOS condition. Shown in Figure 7 is the fitted distribution of the mean excess delay and RMS delay spread using Matlab *dfittool* in indoor environment. It is found that the two kinds of features can be approximately modeled by log-normal PDF (Probability Distribution Function) with different mean and variance.

(2)  Waveform characteristics

**Figure 8:**
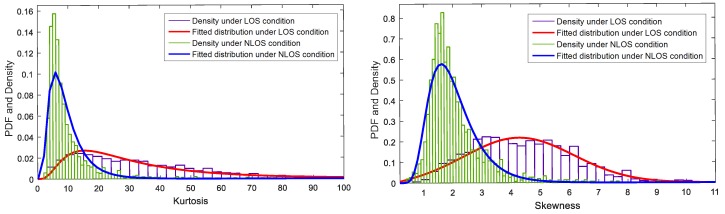
PDF of the kurtosis and skewness.

The kurtosis *k* and skewness *s* are two main waveform statistics to characterise the tailedness or normality and asymmetry of a distribution. The kurtosis and skewness can be given by

(15) $$k=\frac{E[{\left(r-\mu_{r}\right)}^{4}]}{\sigma_{r}^{4}},\;s=\frac{E[{\left(r-\mu_{r}\right)}^{3}]}{\sigma_{r}^{3}},$$

where *r* is the uniform sampling result of relative channel gain and delay, and the size of *r* is equal to ${\hat{\tau }}_{i}$; E[·] is the mathematical expectation operator; and $\mu_{r}$ and $\sigma_{r}$ are the mean and standard deviation of *r*. From Figure 5 and Figure 6, we can see that the waveforms have a bad normality and asymmetry under the LOS condition. Then, *k* and *s* under the LOS condition are larger than those under the NLOS condition. The distribution is shown in Figure 8. The two kinds of features can be approximately modeled by a log-normal PDF, except that the skewness under the LOS condition can be modeled by Rician distribution. The mean and standard deviation of PDF under the NLOS condition are smaller than those under the LOS condition.

(3)  Rician K-factor

**Figure 9:**
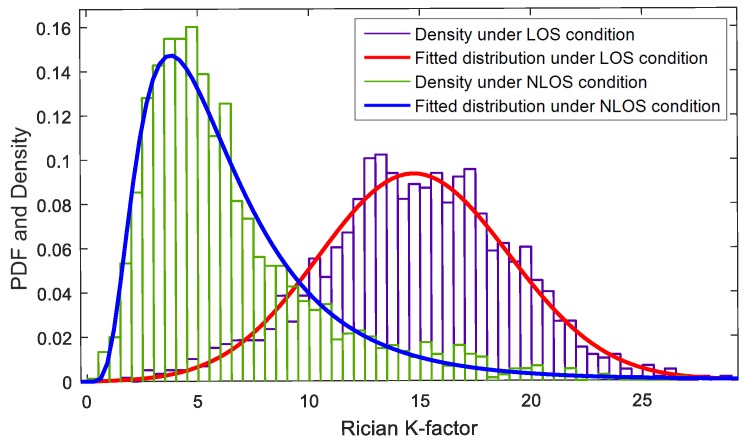
PDF of the Rician K-factor.

The Rician K-factor is the ratio of the LOS component to the diffusion component, and has been widely studied in link quality estimation of wireless communications since it is widely accepted that the unshadowed channel, LOS propagation path, is a Rician fading channel while the shadowed channel, NLOS path, is a Rayleigh fading channel [27,28]. Even though there are many differences between a radio channel and an acoustic channel, the idea about the ratio of the LOS component to the diffuse component is a valuable insight to extract the feature, Rician-K factor, which is denoted by $K_{R}$ and expressed as [27]

(16) $$K_{R}=10\log_{10}\left(\frac{k_{d}^{2}}{2\sigma^{2}}\right),$$

where $k_{d}$ is the strength of the LOS component and σ is the standard deviation of the diffusion path. In wireless communications, if $k_{d}$ is very small and approximates to zero, that means the LOS path is blocked, then $K_{R}=-\infty dB$ and the channel could be described as the Rayleigh fading channel. However, there is no clear evidence that the acoustic channel also follows those two fading distributions. To calculate the Rician K-factor of an acoustic channel, we use $k_{d}=r_{1}$ and $\sigma =\sigma_{r}$. The distribution of the Rician K-factor is shown in Figure 9. The PDF of the Rician K-factor under the NLOS condition could be approximately modeled by a log-normal distribution, while that under the LOS condition could be modeled by a Rician distribution.

(4)  Frequency characteristics of relative channel gain

**Figure 10:**
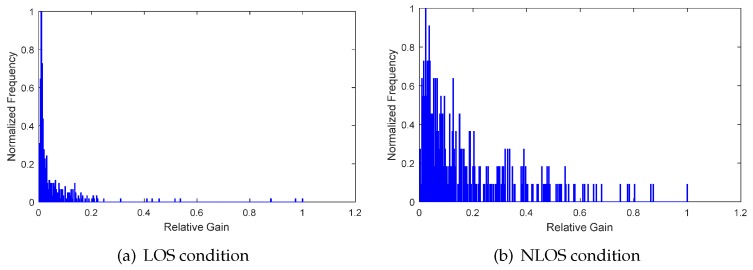
The frequency of relative channel gain in an office room environment.

**Figure 11:**
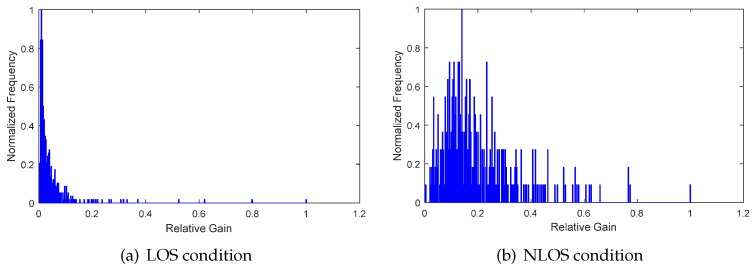
The frequency of relative channel gain in a lobby environment.

**Figure 12:**
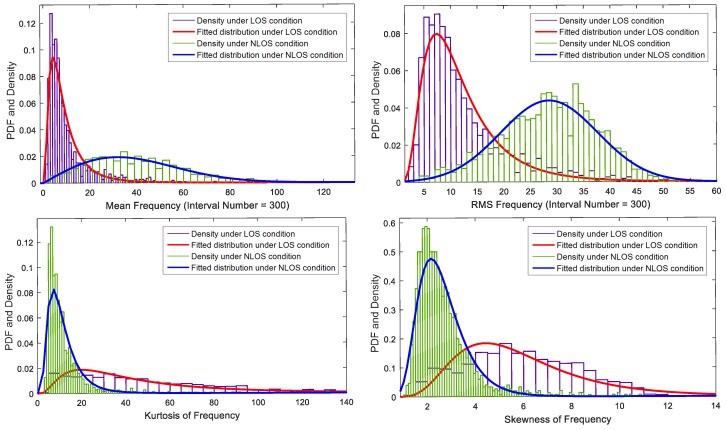
PDFs of the mean, RMS, kurtosis and skewness of frequency.

From the amplitude components of relative channel gain, we can clearly see the difference between LOS and NLOS conditions. By discarding the time delay information and compiling the statistics of the frequency of relative channel gain, we can obtain the frequency distribution, that is the histogram. Shown in Figure 10 and Figure 11 are the frequency distributions of relative channel gain in an office room and lobby environment, respectively. From the waveform of frequency distribution, the features of amplitude characteristics and waveform characteristics are studied by referring to the method of relative channel gain and delay. The mean frequency $g_{m}$ and RMS frequency $g_{rms}$ of relative channel gain frequency are given by:

(17) $$g_{m}=\frac{\sum_{j=1}^{n}\lambda_{j}^{2}f_{j}^{2}}{\sum_{j=1}^{n}\lambda_{j}^{2}},\;g_{rms}=\sqrt{\frac{\sum_{j=1}^{n}\lambda_{j}^{2}f_{j}^{2}}{\sum_{j=1}^{n}\lambda_{j}^{2}}-g_{m}^{2}}\;,$$

where $\lambda_{j},j=1,2,...,n$ is the upper boundary of the *j*th interval and $f_{j}$ is the frequency of relative channel gain amplitude falling into the *j*th interval. During the practical calculation process, $\lambda_{j}=j/n$, since the amplitude of relative channel gain has been normalized. The kurtosis and skewness of frequency distribution are given by:

(18) $$k_{f}=\frac{E[{\left(f-\mu_{f}\right)}^{4}]}{\sigma_{f}^{4}},\;s_{f}=\frac{E[{\left(f-\mu_{f}\right)}^{3}]}{\sigma_{f}^{3}}\;,$$

where $f=\{f_{j}\},j=1,2,...,n$ is the frequency series. As shown in Figure 12, the distributions of $g_{m}$, $g_{rms}$, $k_{f}$ and $s_{f}$ have similar characteristics of $\tau_{med}$, $\tau_{rms}$, *k* and *s*. The feature, like the Rician K-factor, has no physical meaning in the frequency distribution of relative channel gain, since the time delay information is discarded. Thus, this kind of feature has not been studied in this paper.

For the indoor environment, most features also can be approximately modeled by the log-normal PDF, while the skewness, Rician K-factor and RMS frequency of relative channel gain under the LOS condition can be well modeled by the Rician PDF. At the same time, we can clearly observe that the PDFs of these features in indoor environment are quite distinct between the LOS condition and the NLOS condition. This implies that the nine features, which are the mean excess delay $\tau_{med}$, RMS delay spread $\tau_{rms}$, kurtosis *k*, skewness *s*, Rician K-factor $K_{R}$, mean frequency of relative channel gain $g_{m}$, RMS frequency of relative channel gain $g_{rms}$, frequency kurtosis $k_{f}$ and frequency skewness $k_{s}$, can provide good information for acoustic NLOS identification.

## 5. NLOS Identification Based on SVM Classifiers

Acoustic NLOS identification is a binary classification problem. A joint likelihood ratio test could be used to test if a certain received signal is under the LOS or NLOS condition, through the extracted features [22]. However, it is very difficult to determine the real distribution of these features. In Section 4.2, we try to model the PDF of features using Maltab *dfittool* function, but the result is still not satisfactory. It still needs more statistical approaches and a larger size of data set. Therefore, in this paper, we propose the use of non-parametric machine learning techniques to realize acoustic NLOS identification, or LOS/NLOS classification. This is because they do not require a statistical distribution of features under LOS and NLOS conditions, and can perform this binary classification under a common framework.

### 5.1. The SVM Classifier and Kernel Function

Support vector machine (SVM) learning is a supervised learning technique used both for classification and regression problems [29], and has been widely used in many areas. The basic idea of SVM learning is to find the optimal hyperplane as a decision surface which could correctly separate the majority of the data points while maximizing the margins from the hyperplane to each class [30]. For the binary classification problem of acoustic NLOS identification, the audio signals are classified into two classes: positive class and negative class. Acoustic signals received from the NLOS propagation path belong to the positive class with the class label $y^{\left(i\right)}=1$ , while those received from the LOS propagation path belong to the negative class which is denoted by the class label $y^{\left(i\right)}=-1$. In the case that the two classes can be separated, the SVM determines the separating hyperplane which maximizes the margin between the two classes. This is a kind of regression problem to determine the weight vector and bias based on the training set $\left\{(x^{\left(i\right)},y^{\left(i\right)});i=1,...,m\right\}$, where the superscript (i) is the index of the training set; $x^{\left(i\right)}\in R^{n}$ and $y^{\left(i\right)}\in \left\{-1,+1\right\}$ are the features and labels, respectively.

However, the training data collected in the real world usually cannot be separated without error or with small error. In 1995, Cortes and Vapnik introduced the principle of the kernel method to address the separability of features. The kernel function is used for implicitly mapping the input feature vector into an arbitrary high-dimensional feature space that can be linearly separable, because the probability that the feature space could be linearly separated becomes higher through nonlinearly mapping this low-dimensional feature space into a high-dimensional space. Then, in [29], the above mentioned maximization problem is equal to an optimal problem which can be formulated as

(19) $$\min_{w,\xi_{i}}\;J\left(w,\xi \right)=w^{T}w+C\sum_{i=1}^{m}\xi_{i}$$

$$\begin{array}{c} s.t.\;\;y^{\left(i\right)}\left[w\phi \left(x^{\left(i\right)}\right)+b\right]\geq 1-\xi_{i}\\ \;\;\;\;\;\;\;\;\xi_{i}\geq 0,i=1,2,...,m\;, \end{array}$$

where *w* is the weight vector, *b* is a bias, and *T* is the transverse operator, ϕ(·) is the mapping function; the variable $\xi_{i}$ is the positive slack variable that allows the SVM to tolerate misclassification; *C* is a margin parameter which controls the trade-off between minimizing training errors and modelling complexity. Through $\phi (x^{\left(i\right)})$, the input feature vector $x^{\left(i\right)}$ is mapped from the low-dimensional feature space $R^{n}$ into a higher dimensional feature space *S*. Thus, according to the Lagrangian principle, its corresponding dual problem is

(20) $$\min_{\alpha }\frac{1}{2}\alpha^{T}Q\alpha -e^{T}\alpha$$

$$\begin{array}{c} s.t.\;\;\;\;\;\;0\leq \alpha \leq C,\;\;\;\;\;\;\;\;\;\;\;\;y^{T}\alpha =0, \end{array}$$

where α is the vector of the Lagrange multiplier, $e={\left[1,1,....,1\right]}^{T}$, *Q* is an *m* by *m* positive semi-definite matrix which is given by

(21) $$Q_{ij}=y^{\left(i\right)}y^{\left(j\right)}K(x^{\left(i\right)},x^{\left(j\right)}),$$

where $K\left(x^{\left(i\right)},x^{\left(j\right)}\right)=\phi {\left(x^{\left(i\right)}\right)}^{T}\phi (x^{\left(j\right)})$ is known as the kernel function, which is an inner product of mapping function ϕ(·). In other words, the computation of the kernel method becomes possible in high-dimensional space, because it computes the inner product as a direct function of input space without explicitly computing the mapping [31]. Then, by using the kernel method, the discriminant function of the SVM classifier is a function $R^{n}\to \left\{-1,+1\right\}$ with the form of

(22) $$y\left(x\right)=\mathrm{sgn}\left[\sum_{i=1}^{m}y_{i}\alpha_{i}K\left(x_{i,}x\right)+b\right]\;,$$

where $K\left(x^{\left(i\right)},x\right)=\phi {\left(x^{\left(i\right)}\right)}^{T}\phi (x)$. Generally, the widely used kernel functions mainly include a radial-based function (RBF) kernel $K_{rbf}\left(\cdot \right)$, a polynomial kernel $K_{p}\left(\cdot \right)$, a linear kernel $K_{l}\left(\cdot \right)$ and a sigmoid kernel $K_{s}\left(\cdot \right)$. These kernel functions are expressed as

(23) $$\left\{\begin{array}{c} K_{rbf}\left(x^{\left(i\right)},x\right)=e^{\left(-\gamma {∥x^{\left(i\right)}-x∥}^{2}\right)}\\ K_{p}\left(x^{\left(i\right)},x\right)={\left(\gamma \langle x^{\left(i\right)},x\rangle +c\right)}^{d}\\ K_{l}\left(x^{\left(i\right)},x\right)=\langle x^{\left(i\right)},x\rangle \\ K_{s}\left(x^{\left(i\right)},x\right)=\tanh\left(\gamma \langle x^{\left(i\right)},x\rangle +c\right) \end{array}\right.,$$

where γ and *c* are the positive kernel coefficients and *d* is the degree of polynomial kernel. Generally, we choose γ=1, c=0 and d=2. In this paper, the four kinds of kernel functions are tested individually, and the kernel with the best performance is selected as the kernel function for acoustic NLOS identification. Furthermore, to evaluate the best performance of the SVM classifier with the chosen kernel function, the dimension of feature space is selected from 1 to 9. In addition, different feature combinations are also tested to determine the best feature combination, which is chosen from the feature set $x^{\left(i\right)}\in \left[\tau_{med}^{\left(i\right)},\tau_{rms}^{\left(i\right)},k^{\left(i\right)},s^{\left(i\right)},K_{R}^{\left(i\right)},g_{m}^{\left(i\right)},g_{rms}^{\left(i\right)},k_{f}^{\left(i\right)},s_{f}^{\left(i\right)}\right],i=1,2,...,m$.

### 5.2. Cross-Validation and Evaluation Criteria

In order to evaluate the performance of classifiers, a K-fold cross-validation process (K=10) is carried out to evaluate the performance of SVM classifiers with each kernel. Firstly, all the collected acoustic signals are mixed together as a whole data set and randomly divided into 10 non-overlapping subsets with the same data size. Secondly, any possible combination of nine subsets, that is $C_{10}^{9}$, is selected from the 10 non-overlapping subsets as the training set for the estimation of the parameters in the SVM classifier, and the rest are used for the validation set, which is also called the testing set. Through repeating the above process 10 times, each subset is tested as a validation set. Furthermore, the cross-validation procedure is repeated 10 times, and the evaluated performance of the classifier is calculated by averaging the results under each kind of evaluation criterion.

The widely used evaluation criteria in binary classification include accuracy, error rate, sensitivity, specificity, precision, recall ratio, and F1-Measure [32]. In this paper, accuracy, precision and F1-Measure are selected, since they are easy to be computed and understood by humans. The accuracy metric measures the ratio of correct predictions over the total number of data evaluated. Under this criterion, we can comprehensively evaluate a feature in each classifier. The precision metric focuses on how many returned positive results are correctly classified in a positive class which is predicted as positive during the classification process. F1-Measure is a measure of a test’s accuracy and considers both the precision and the recalled metrics. Paper [33] reported that the F1-Measure metric was more accurate at optimizing a classifier for binary classification. We use the accuracy criterion to evaluate the performance of each kernel while the results of precision and F1-Measure are also listed. The accuracy, precision and F1-Measure can be, respectively, given by

(24) $$\left\{\begin{array}{c} \mathrm{accuracy}=\frac{t_{p}+t_{n}}{t_{p}+t_{n}+f_{p}+f_{n}}\\ \mathrm{precision}=\frac{t_{p}}{t_{p}+f_{p}}\\ F1-\mathrm{Measure}=\frac{2t_{p}}{2t_{p}+t_{n}+f_{p}} \end{array}\right.,$$

where $t_{p}$ and $t_{n}$ denote the number of misclassified negative and positive data, respectively. Meanwhile, $f_{p}$ and $t_{n}$ denote the number of misclassified negative and positive data, respectively [32].

### 5.3. Test Results and Discussion

In order to choose a kernel function for the SVM classifier, the classification performance of four kinds of kernel functions are tested based on the data set with more than 10 thousand acoustic signals collected in indoor environment. The classifiers are tested in a different feature set $F^{M}$, where *M* is the size of the feature set. Due to the maximum feature set size in this paper being 9, that is M=1,2,...,9, it is possible for us to test the performance of classifiers in each feature set by using the brute-force method. For the feature set size *M*, the number of feature sets with different feature combinations is $C_{9}^{M}$. M=1 means using the feature set with one kind of feature to evaluate the availability of features proposed in this paper. The test results are presented in Table 1.

In Table 1, we are especially concerned with the performance under the accuracy criterion, while the results under precision and F1-Measure are also listed. The mean accuracy and median accuracy are calculated and listed below the table for each kind of kernel function. The results show that the sigmoid kernel function has the lowest classification performance among the four kinds of kernel function. The performances of the other three kernel functions are close to each other. The accuracy of the SVM classifier with the RBF kernel, polynomial kernel and linear kernel is between 76% and 87% when solely one feature of the nine is used. Meanwhile, the mean accuracy is around 83%, the median accuracy is around 84%, and the best feature is the mean frequency $g_{m}$. Then, we can conclude that the nine features extracted from the received signals are available for NLOS identification by using an SVM classifier with three kernel functions, and could achieve a high accuracy and stability. This proves that the relative channel gain and delay estimation approach proposed in Section 4.2 can effectively support the feature extraction.

From Table 1, the SVM classifier with RBF kernel function has the best classification accuracy. However, the optimal kernel function still cannot be determined, due to the small performance gaps between the RBF kernel, polynomial kernel and linear kernel. To select the optimal kernel function of the SVM classifier for acoustic NLOS identification, the performance of the SVM classifier with the three kernel functions is individually tested in the feature data $F^{M}$ with the size of M=1,2,...,9, and the test results are presented in Table 2 under the evaluation criterion of accuracy. The feature combinations, which could achieve the highest classification accuracy in each feature set size, are listed for each kind of kernel function, respectively, corresponding to its accuracy test result. The average accuracy in each feature set size is also listed at the right side of the table. The best feature set and the best feature combination for each kind of kernel function are listed below the table.

**Figure 13:**
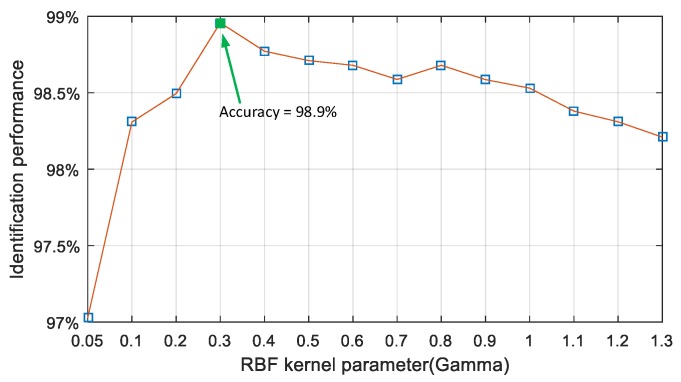
Selection of the optimal RBF kernel parameter γ.

From Table 2, through the comparison of the test results of the three kernel functions, it can be found that mapping the nine features extracted from the indoor acoustic signals through RBF kernel function yields a better result than polynomial and linear kernels. That means that the input feature vectors are nonlinearly mapped into a higher dimensional space and become more linearly separable, by using the RBF kernel function. Thus, the optimal kernel function of the SVM classifier is the RBF kernel for acoustic NLOS identification, where the mean accuracy is 96.2% and median accuracy is 98.3%. The best feature set size is M=5 with the best feature combination $\left\{k,g_{m},g_{rms},k_{f},s_{f}\right\}$, which supports the SMV classifier to achieve a 98.5% identification accuracy. The performances of the SVM classifier with the polynomial kernel and linear kernel are close to each other, with the mean accuracy being 88.7% and median accuracy being 89%. Meanwhile, by comparing the best, worst and average accuracy of each kind of feature combination, it is also easy to find that the performance of each kind of classifier using each kind of feature combination has a high stability. Furthermore, the time consumption of a single identification is from 95ms to 100ms, which is counted by the *tic* and *toc* function of Maltab. Consequently, this classifier can be implemented in practical real-time applications. To optimize the γ value of RBF kernel function, the relationship between identification performance and γ is plotted in Figure 13, and the SVM with the RBF kernel with γ=0.3 has the best identification result (98.9%) according to Figure 13, and the best feature set size is M=6 with the best feature combination $F^{6}=\left\{\tau_{med},\tau_{rms},k,s,K_{R},g_{m}\right\}$.

To further investigate the performance of the SVM classifier with RBF kernel function for acoustic NLOS identification, the performances of traditional classifiers based on logistic regression (LR) [34] and linear discriminant analysis (LDA) [35] are tested under the same cross-validation method, and the results are presented in Table 3. Comparing the results of Table 2 and Table 3, we can see that the performance of LR and LDA classifiers is close to the SVM classifier with the polynomial kernel and linear kernel. In general, the overall performance of the SVM with the RBF kernel is better than the LR and LDA approaches for acoustic NLOS identification.

## 6. Conclusions

In this paper, we focus on acoustic NLOS identification for smartphone indoor localization and propose an approach based on acoustic channel characteristics. Through analyzing indoor acoustic propagation, the changes of acoustic channel from the LOS condition to the NLOS condition are characterized as the difference of channel gain and delay between the two propagation scenarios. Then, in order to mitigate the Doppler Effect and reduce the computational complexity, an efficient approach to estimate relative channel gain and delay based on the cross-correlation method is proposed. Nine novel features have been extracted based on time delay characteristics, waveform characteristics, Rician K-factor and frequency characteristics of relative channel gain.

To realize acoustic NLOS identification, an SVM classifier with four kinds of kernel functions has been proposed. By using the accuracy metric as an evaluation criterion, the evaluation result shows that the optimal kernel function is the RBF kernel. At the same time, the comparison results between the SVM and the traditional classifiers based on LR and LDA show that the SVM with the RBF kernel function method is the optimal classifier for acoustic NLOS identification. Meanwhile, we can conclude that (1) using acoustic channel characteristics for indoor localization is an efficient way to realize acoustic NLOS identification; (2) the features extracted from the received signals are available for NLOS identification and could achieve high accuracy and stability; (3) the channel parameter estimation approach proposed in this paper could effectively support the feature extraction.

## Figures and Tables

**Table 1:** The performance of four kinds of kernel functions in $F^{1}$.

**RBF Kernel Function**				**Polynomial Kernel Function**			
Feature	Precision	Accuracy	F1-Measure	Feature	Precision	Accuracy	F1-Measure
$\tau_{med}$	0.818	0.826	0.850	$\tau_{med}$	0.832	0.832	0.850
$\tau_{rms}$	0.749	0.781	0.824	$\tau_{rms}$	0.776	0.770	0.795
*k*	0.837	0.823	0.841	*k*	0.784	0.811	0.840
*s*	0.840	0.828	0.846	*s*	0.803	0.821	0.844
$K_{R}$	0.896	0.853	0.864	$K_{R}$	0.895	0.858	0.873
$g_{m}$	0.858	0.867	0.885	$g_{m}$	0.883	0.858	0.871
$g_{rms}$	0.850	0.851	0.870	$g_{rms}$	0.848	0.837	0.854
$k_{f}$	0.838	0.852	0.872	$k_{f}$	0.813	0.847	0.871
$s_{f}$	0.838	0.849	0.870	$s_{f}$	0.827	0.846	0.868
Mean accuracy		0.837		Mean accuracy		0.831	
Median accuracy		0.849		Median accuracy		0.837	
Best feature		$g_{m}$		Best feature		$g_{m}$	
**Linear Kernel Function**				**Sigmoid Kernel Function**			
Feature	Precision	Accuracy	F1-Measure	Feature	Precision	Accuracy	F1-Measure
$\tau_{med}$	0.825	0.826	0.848	$\tau_{med}$	0.564	0.564	0.721
$\tau_{rms}$	0.783	0.763	0.789	$\tau_{rms}$	0.559	0.559	0.717
*k*	0.778	0.800	0.834	*k*	0.566	0.566	0.723
*s*	0.813	0.819	0.846	*s*	0.289	0.205	0.290
$K_{R}$	0.876	0.849	0.862	$K_{R}$	0.512	0.456	0.625
$g_{m}$	0.884	0.861	0.874	$g_{m}$	0.549	0.549	0.709
$g_{rms}$	0.859	0.852	0.869	$g_{rms}$	0.559	0.559	0.717
$k_{f}$	0.810	0.844	0.870	$k_{f}$	0.544	0.544	0.705
$s_{f}$	0.827	0.847	0.868	$s_{f}$	0.397	0.297	0.430
Mean accuracy		0.829		Mean accuracy		0.478	
Median accuracy		0.844		Median accuracy		0.549	
Best feature		$g_{m}$		Best feature		*k*	

**Table 2:** The performance of three kinds of kernel functions under the accuracy criterion in $F^{M}$.

**SVM with RBF Kernel Function**				
Best		Worst		Average
Feature combination	Accuracy	Feature combination	Accuracy	
$F^{1}=\left\{g_{m}\right\}$	0.867	$F^{1}=\left\{\tau_{rms}\right\}$	0.781	0.837
$F^{2}=\left\{K_{R},g_{m}\right\}$	0.913	$F^{2}=\left\{k,s\right\}$	0.841	0.877
$F^{3}=\left\{k,K_{R},g_{m}\right\}$	0.975	$F^{3}=\left\{s,k_{f},s_{f}\right\}$	0.864	0.931
$F^{4}=\left\{\tau_{med},\tau_{rms},K_{R},g_{m}\right\}$	0.984	$F^{4}=\left\{s,g_{rms},k_{f},s_{f}\right\}$	0.902	0.967
$F^{5}=\left\{\tau_{med},\tau_{rms},k,g_{m},g_{rms}\right\}$	0.985	$F^{5}=\left\{k,s,g_{rms},k_{f},s_{f}\right\}$	0.952	0.980
$F^{6}=\left\{\tau_{med},\tau_{rms},s,g_{m},g_{rms},s_{f}\right\}$	0.984	$F^{6}=\left\{k,s,K_{R},g_{rms},k_{f},s_{f}\right\}$	0.980	0.982
$F^{7}=\left\{\tau_{rms},s,K_{R},g_{m},g_{rms},k_{f},s_{f}\right\}$	0.983	$F^{7}=\left\{\tau_{rms},k,s,K_{R},g_{rms},k_{f},s_{f}\right\}$	0.981	0.982
$F^{8}=\left\{\tau_{med},k,s,K_{R},g_{m},g_{rms},k_{f},s_{f}\right\}$	0.983	$F^{8}=\left\{\tau_{med},\tau_{rms},k,s,K_{R},g_{m},g_{rms},s_{f}\right\}$	0.981	0.982
$F^{9}=\left\{\tau_{med},\tau_{rms},k,s,K_{R},g_{m},g_{rms},k_{f},s_{f}\right\}$	0.983	$F^{9}=\left\{\tau_{med},\tau_{rms},k,s,K_{R},g_{m},g_{rms},k_{f},s_{f}\right\}$	0.983	0.983
Mean accuracy	0.962			
Median accuracy	0.983			
Best feature combination $F^{5}=\left\{\tau_{med},\tau_{rms},k,g_{m},g_{rms}\right\}$				
**SVM with Polynomial Kernel Function**				
Best		Worst		Average
Feature combination	Accuracy	Feature combination	Accuracy	
$F^{1}=\left\{g_{m}\right\}$	0.858	$F^{1}=\left\{\tau_{rms}\right\}$	0.770	0.831
$F^{2}=\left\{K_{R},g_{m}\right\}$	0.873	$F^{2}=\left\{\tau_{med},\tau_{rms}\right\}$	0.827	0.853
$F^{3}=\left\{\tau_{med},K_{R},g_{m}\right\}$	0.886	$F^{3}=\left\{\tau_{med},\tau_{rms},k_{f}\right\}$	0.830	0.860
$F^{4}=\left\{\tau_{med},K_{R},g_{m},k_{f}\right\}$	0.889	$F^{4}=\left\{\tau_{med},\tau_{rms},k,k_{f}\right\}$	0.842	0.863
$F^{5}=\left\{K_{R},g_{m},g_{rms},k_{f},s_{f}\right\}$	0.890	$F^{5}=\left\{\tau_{med},\tau_{rms},k,s,s_{f}\right\}$	0.843	0.868
$F^{6}=\left\{\tau_{med},s,K_{R},g_{m},g_{rms},k_{f}\right\}$	0.895	$F^{6}=\left\{\tau_{med},\tau_{rms},k,g_{rms},k_{f},s_{f}\right\}$	0.848	0.873
$F^{7}=\left\{\tau_{med},\tau_{rms},s,K_{R},g_{m},k_{f},s_{f}\right\}$	0.896	$F^{7}=\left\{\tau_{med},\tau_{rms},k,s,g_{rms},k_{f},s_{f}\right\}$	0.853	0.880
$F^{8}=\left\{\tau_{med},\tau_{rms},k,s,K_{R},g_{m},k_{f},s_{f}\right\}$	0.903	$F^{8}=\left\{\tau_{med},\tau_{rms},k,s,g_{m},g_{rms},k_{f},s_{f}\right\}$	0.866	0.891
$F^{9}=\left\{\tau_{med},\tau_{rms},k,s,K_{R},g_{m},g_{rms},k_{f},s_{f}\right\}$	0.892	$F^{9}=\left\{\tau_{med},\tau_{rms},k,s,K_{R},g_{m},g_{rms},k_{f},s_{f}\right\}$	0.892	0.892
Mean accuracy	0.887			
Median accuracy	0.890			
Best feature combination $F^{8}=\left\{\tau_{med},\tau_{rms},k,s,K_{R},g_{m},k_{f},s_{f}\right\}$				
**SVM with Linear Kernel Function**				
Best		Worst		Average
Feature combination	Accuracy	Feature combination	Accuracy	
$F^{1}=\left\{g_{m}\right\}$	0.861	$F^{1}=\left\{\tau_{rms}\right\}$	0.763	0.829
$F^{2}=\left\{K_{R},g_{m}\right\}$	0.876	$F^{2}=\left\{\tau_{med},\tau_{rms}\right\}$	0.825	0.853
$F^{3}=\left\{\tau_{rms},K_{R},g_{m}\right\}$	0.884	$F^{3}=\left\{\tau_{med},\tau_{rms},k\right\}$	0.828	0.859
$F^{4}=\left\{\tau_{med},K_{R},g_{m},k_{f}\right\}$	0.887	$F^{4}=\left\{\tau_{med},\tau_{rms},k,k_{f}\right\}$	0.843	0.864
$F^{5}=\left\{\tau_{med},\tau_{rms},K_{R},g_{m},k_{f}\right\}$	0.890	$F^{5}=\left\{\tau_{med},\tau_{rms},g_{rms},k_{f}\right\}$	0.840	0.867
$F^{6}=\left\{\tau_{med},\tau_{rms},s,K_{R},g_{m},s_{f}\right\}$	0.895	$F^{6}=\left\{\tau_{med},\tau_{rms},k,s,g_{rms},s_{f}\right\}$	0.842	0.873
$F^{7}=\left\{\tau_{med},\tau_{rms},k,K_{R},g_{m},k_{f},s_{f}\right\}$	0.896	$F^{7}=\left\{\tau_{rms},k,s,g_{m},g_{rms},k_{f},s_{f}\right\}$	0.852	0.878
$F^{8}=\left\{\tau_{med},\tau_{rms},k,s,K_{R},g_{m},k_{f},s_{f}\right\}$	0.902	$F^{8}=\left\{\tau_{med},\tau_{rms},k,s,g_{m},g_{rms},k_{f},s_{f}\right\}$	0.863	0.887
$F^{9}=\left\{\tau_{med},\tau_{rms},k,s,K_{R},g_{m},g_{rms},k_{f},s_{f}\right\}$	0.894	$F^{9}=\left\{\tau_{med},\tau_{rms},k,s,K_{R},g_{m},g_{rms},k_{f},s_{f}\right\}$	0.894	0.894
Mean accuracy	0.887			
Median accuracy	0.890			
Best feature combination $F^{8}=\left\{\tau_{med},\tau_{rms},k,s,K_{R},g_{m},k_{f},s_{f}\right\}$				

**Table 3:** The performance of logistic regression (LR) and the linear discriminant analysis (LDA) classifier under the accuracy criterion in $F^{M}$.

**Logistic Regression**				
Best		Worst		Average
Feature combination	Accuracy	Feature combination	Accuracy	
$F^{1}=\left\{g_{m}\right\}$	0.860	$F^{1}=\left\{\tau_{rms}\right\}$	0.776	0.830
$F^{2}=\left\{K_{R},g_{m}\right\}$	0.882	$F^{2}=\left\{\tau_{med},\tau_{rms}\right\}$	0.803	0.850
$F^{3}=\left\{s,K_{R},g_{m}\right\}$	0.882	$F^{3}=\left\{\tau_{med},\tau_{rms},s\right\}$	0.828	0.858
$F^{4}=\left\{k,K_{R},g_{m},g_{rms}\right\}$	0.893	$F^{4}=\left\{\tau_{rms},k,s,s_{f}\right\}$	0.837	0.862
$F^{5}=\left\{s,K_{R},g_{m},g_{rms},s_{f}\right\}$	0.889	$F^{5}=\left\{\tau_{med},\tau_{rms},s,g_{rms},k_{f}\right\}$	0.839	0.866
$F^{6}=\left\{\tau_{med},K_{R},g_{m},g_{rms},k_{f},s_{f}\right\}$	0.903	$F^{6}=\left\{\tau_{rms},k,s,g_{rms},k_{f},s_{f}\right\}$	0.839	0.874
$F^{7}=\left\{\tau_{med},\tau_{rms},s,K_{R},g_{m},k_{f},s_{f}\right\}$	0.895	$F^{7}=\left\{\tau_{med},\tau_{rms},k,s,g_{rms},k_{f},s_{f}\right\}$	0.839	0.878
$F^{8}=\left\{\tau_{med},\tau_{rms},k,s,K_{R},g_{m},g_{rms},s_{f}\right\}$	0.895	$F^{8}=\left\{\tau_{med},\tau_{rms},k,s,K_{R},g_{rms},k_{f},s_{f}\right\}$	0.877	0.886
$F^{9}=\left\{\tau_{med},\tau_{rms},k,s,K_{R},g_{m},g_{rms},k_{f},s_{f}\right\}$	0.890	$F^{9}=\left\{\tau_{med},\tau_{rms},k,s,K_{R},g_{m},g_{rms},k_{f},s_{f}\right\}$	0.890	0.890
Mean accuracy	0.888			
Median accuracy	0.890			
Best feature combination $F^{6}=\left\{\tau_{med},K_{R},g_{m},g_{rms},k_{f},s_{f}\right\}$				
**LDA**				
Best		Worst		Average
Feature combination	Accuracy	Feature combination	Accuracy	
$F^{1}=\left\{K_{R}\right\}$	0.848	$F^{1}=\left\{\tau_{rms}\right\}$	0.760	0.809
$F^{2}=\left\{K_{R},g_{m}\right\}$	0.882	$F^{2}=\left\{\tau_{med},\tau_{rms}\right\}$	0.767	0.844
$F^{3}=\left\{\tau_{rms},s,K_{R}\right\}$	0.879	$F^{3}=\left\{\tau_{med},\tau_{rms},s\right\}$	0.829	0.855
$F^{4}=\left\{\tau_{rms},s,K_{R},k_{f}\right\}$	0.878	$F^{4}=\left\{\tau_{med},\tau_{rms},k,k_{f}\right\}$	0.834	0.860
$F^{5}=\left\{\tau_{med},\tau_{rms},s,K_{R},g_{m}\right\}$	0.887	$F^{5}=\left\{\tau_{med},\tau_{rms},k,k_{f},s_{f}\right\}$	0.836	0.864
$F^{6}=\left\{\tau_{med},\tau_{rms},s,K_{R},g_{m},g_{rms}\right\}$	0.891	$F^{6}=\left\{\tau_{med},\tau_{rms},k,s,k_{f},s_{f}\right\}$	0.847	0.867
$F^{7}=\left\{\tau_{med},\tau_{rms},k,s,K_{R},g_{m},k_{f}\right\}$	0.889	$F^{7}=\left\{\tau_{med},\tau_{rms},s,g_{m},g_{rms},k_{f},s_{f}\right\}$	0.848	0.870
$F^{8}=\left\{\tau_{med},k,s,K_{R},g_{m},g_{rms},k_{f},s_{f}\right\}$	0.885	$F^{8}=\left\{\tau_{med},\tau_{rms},k,s,g_{m},g_{rms},k_{f},s_{f}\right\}$	0.855	0.874
$F^{9}=\left\{\tau_{med},\tau_{rms},k,s,K_{R},g_{m},g_{rms},k_{f},s_{f}\right\}$	0.873	$F^{9}=\left\{\tau_{med},\tau_{rms},k,s,K_{R},g_{m},g_{rms},k_{f},s_{f}\right\}$	0.873	0.873
Mean accuracy	0.879			
Median accuracy	0.882			
Best feature combination $F^{7}=\left\{\tau_{med},\tau_{rms},k,s,K_{R},g_{m},k_{f}\right\}$				
